# Concave and convex growth do not differ over tethered vertebral segments, even with open tri-radiate cartilage

**DOI:** 10.1007/s43390-023-00683-0

**Published:** 2023-04-01

**Authors:** Daniel Farivar, Stefan Parent, Firoz Miyanji, Michael J. Heffernan, Ron El-Hawary, A. Noelle Larson, Lindsay M. Andras, Pediatric Spine Study Group, David L. Skaggs

**Affiliations:** 1 Cedars-Sinai Spine, 444 S San Vicente Blvd, Ste 901, Los Angeles, CA 90048 USA; 2 Department of Surgery, Université de Montréal, Montreal, QC Canada; 3 Department of Orthopedics, BC Children’s Hospital, Vancouver, BC Canada; 4 Children’s Orthopaedic Center, Children’s Hospital Los Angeles, Los Angeles, CA USA; 5 Orthopedics, Izaak Walton Killam (IWK) Health Centre, Halifax, Canada; 6 Department of Orthopedic Surgery, Mayo Clinic, 200 First Street SW, Rochester, MN 55905 USA

**Keywords:** Pediatric spine, Vertebral body tethering, Growth modulation, Idiopathic scoliosis

## Abstract

**Purpose:**

To assess the following hypotheses related to vertebral body tethering (VBT): 1. VBT is associated with asymmetric (concave > convex) increases in height over the instrumented vertebra. 2. The instrumented Cobb angle improves following VBT surgery with growth.

**Methods:**

This is a retrospective case series of pediatric patients from a multicenter scoliosis registry treated with VBT between 2013 to 2021. Inclusion criteria: patients with standing radiographs at < 4 months and ≥ 2 years after surgery. Distances between the superior endplate of the UIV and the inferior endplate of the LIV were measured at the concave corner, mid-point, and convex corner of the endplates. The UIV-LIV angle was recorded. Subgroup analyses included comparing different Risser scores and tri-radiate cartilage (TRC) closed versus open using student t-tests.

**Results:**

83 patients met inclusion criteria (92% female; age at time of surgery 12.5 ± 1.4 years) with mean follow-up time of 3.8 ± 1.4 years. Risser scores at surgery were: 0 (n = 33), 1 (n = 12), 2 (n = 10), 3 (n = 11), 4 (n = 12), and 5 (n = 5). Of the 33 Risser 0 patients, 17 had an open TRC, 16 had a closed TRC. The UIV-LIV distance at concave, middle, and convex points significantly increased from immediate post-op to final-follow-up for Risser 0 patients, but not for Risser 1–5 patients. Increases in UIV-LIV distance were not significantly different between concave, middle, and convex points for all groups. There was no significant improvement or worsening in UIV-LIV angle for any group.

**Conclusion:**

At a mean of 3.8 years following VBT, 33 Risser 0 patients demonstrated significant growth in the instrumented segment, though there was no difference between concave or convex growth, even for patients with open TRC.

## Introduction

Adolescent idiopathic scoliosis (AIS) is a three-dimensional deformity involving lateral deviation of the spine [1]. The goal of treatment is to correct deformity without compromising lung development and spine and chest growth, with the current gold standard being posterior spinal fusion (PSF) [2–4]. Although PSF has a proven track record of good outcomes, it leads to an irreversible stage of permanent spinal fusion, with a chief concern being reduced growth after intervention [5]. This poses a challenge in determining ideal surgical timing. As a result, there has been an increasing interest in “growth-friendly” treatment strategies for AIS, such as vertebral body tethering (VBT).

VBT is a non-fusion procedure for skeletally immature, preadolescent patients to address scoliosis deformity. In 2010, Crawford and Lenke reported the first case successfully managed with VBT in an 8-year old [6]. Since then, multiple case series and clinical trials have been published with encouraging results [7–13]. It is thought to rely on the asymmetric inhibition of vertebral growth by applying the Hueter-Volkmann principle: growth plate compression slows vertebral growth while growth plate distraction promotes vertebral growth [14–19]. In theory, deformity should be improved as tethered vertebra grow. The purpose of this study was to better describe the growth of instrumented vertebra following VBT. We hypothesized that (1) VBT is associated with asymmetric (concave > convex) increases in height over the instrumented vertebra and that (2) the instrumented Cobb angle improves following VBT with growth.

## Methods

This is a retrospective case series of pediatric patients from a multicenter scoliosis registry treated with VBT between 2013 to 2021. Each site obtained institutional review board approval and consent from patients for use of data in future research. The inclusion criteria consisted of all patients less than 17 years of age with idiopathic scoliosis undergoing VBT with standing radiographs at ≤ 4 months after surgery and at ≥ 2 years after surgery. Patients with suspected broken tethers were excluded.

**Fig. 1:**
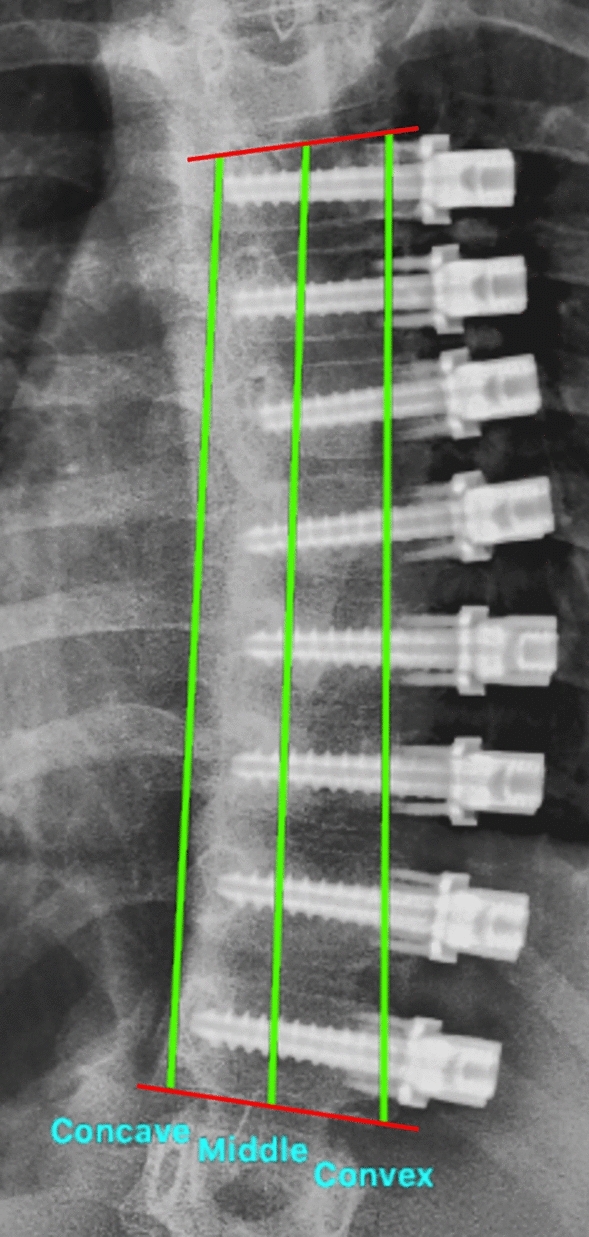
Example of UIV-LIV heights measured from the concave, middle, and convex aspects of instrumented vertebrae (green lines), and the instrumented Cobb angle (red lines)

Demographic data collected included age, sex, number of tethered vertebra, Risser score, and open versus closed tri-radiate cartilage (TRC). On postoperative radiographs the distances between the superior endplate of the upper instrumented vertebrae and inferior endplate of the lower instrumented vertebrae (UIV-LIV distance) were measured at the concave corner, mid-point, and convex corner of the endplates (Fig. 1). The instrumented Cobb angle (UIV-LIV angle) was recorded (Fig. 1). Spine measurements were performed by two authors [initials blinded for peer review] on calibrated images using the Surgimap software (Nemaris Inc., New York, NY). Authors were blinded to patient identifying information and timing of surgery during image review by using a linking list that provided unique codes for each image. At the conclusion of image review, codes were merged with identifying information to allow for data analysis.

## Results

A total of 83 patients met the inclusion criteria (92% female; age at time of surgery 12.5 ± 1.4 years) with a mean follow-up time of 3.8 ± 1.4 years (Table 1). Risser scores at time of surgery were: 0 (n = 33), 1 (n = 12), 2 (n = 10), 3 (n = 11), 4 (n = 12), and 5 (n = 5). Of the 33 Risser 0 patients, 17 had an open TRC while 16 had a closed TRC. At final follow-up, the mean number of tethered vertebral levels was 7.5 ± 0.8.

**Table 1:** Demographic information of study participants

Characteristic	Overall cohort (*N* = 83)
Age at time of surgery [mean ± SD (min–max)]	12.5 ± 1.4 years (8.2–16.4)
Gender [*N* (%)]	
Female	76 (91.5%)
Male	7 (8.4%)
Risser [*N* (%)]	
Risser 0, TRC Open	17 (20.4%)
Risser 0 TRC Closed	16 (19.2%)
Risser 1	12 (14.4%)
Risser 2	10 (12.0%)
Risser 3	11 (13.2%)
Risser 4	12 (14.4%)
Risser 5	5 (6.0%)
Number of tethered vertebral levels [mean ± SD (min–max)]	7.5 ± 0.8 levels (5–11)
Follow-up time [mean ± SD (min–max)]	3.8 ± 1.4 years (2.0–5.8)

*SD* standard deviation, *TRC* tri-radiate cartilage

The UIV-LIV distance at concave, middle, and convex points all significantly increased from immediate post-op to final follow-up for the Risser 0 TRC open and TRC closed patients, but not for Risser 1–5 patients **(**Table 2**).** Increases in UIV-LIV distance were not significantly different between concave and convex points for all skeletal maturity groups. Pre-op UIV-LIV angles were 48.8 ± 9.0° (Risser 0, TRC open), 48.6 ± 9.1° (Risser 0, TRC closed), 51.4 ± 9.1° (Risser 1), and 50.8 ± 9.4° (Risser 2–5). There were no significant changes in UIV-LIV angle in any group between immediate post-op to final follow-up. Risser 0, TRC open patients had a non-significant decrease of 4.0° (22.1 ± 10.3° to 18.1 ± 12.5°) from immediate post-op to final follow-up (p = 0.316). In those with Risser scores 2–5, there was a non-significant increase of 1.9° (24.0 ± 9.0° to 25.9 ± 11.9°) (p = 0.435).

**Table 2:** Changes in (A) UIV-LIV distance and (B) UIV-LIV angle by Risser and status of tri-radiate cartilage

(A) Changes in UIV-LIV Distance			
Average difference in UIV-LIV distance: immediate post-op to final follow-up	Concave	Middle	Convex
Risser 0: TRC open (n = 17) p = 0.449 (concave vs. convex)	+ 15.8 mm (11.9%) (p = 0.009)	+ 15.2 mm (10.5%) (p = 0.014)	+ 12.2 mm (8.1%) (p = 0.048)
Risser 0: TRC closed (n = 16) p = 0.890 (concave vs. convex)	+ 16.8 mm (12.4%) (p = 0.044)	+ 17.0 mm (12.2%) (p = 0.024)	+ 17.6 mm (12.2%) (p = 0.016)
Risser 1 (n = 12) p = 0.884 (concave vs. convex)	+ 10.7 mm (7.6%) (p = 0.169)	+ 10.8 mm (7.4%) (p = 0.169)	+ 10.2 mm (6.7%) (p = 0.197)
Risser 2–5 (n = 38) p = 0.681 (concave vs. convex)	+ 8.9 mm (6.6%) (p = 0.101)	+ 9.8 mm (6.8%) (p = 0.124)	+ 9.9 mm (6.9%) (p = 0.097)

(B) Changes in UIV-LIV Angle			
UIV-LIV angle	Immediate post-op	Final follow-up	Difference
Risser 0: TRC open (n = 17)	22.1 ± 10.3°	18.1 ± 12.5°	− 4.0° (p = 0.316)
Risser 0: TRC closed (n = 16)	22.1 ± 11.0°	22.5 ± 14.3°	+ 0.4° (p = 0.929)
Risser 1 (n = 12)	23.7 ± 9.9°	21.2 ± 13.0°	− 2.5° (p = 0.601)
Risser 2–5 (n = 38)	24.0 ± 9.0°	25.9 ± 11.9°	+ 1.9° (p = 0.435)

*TRC* tri-radiate cartilage

## Discussion

The main findings from this study were that, of all skeletal maturity groups, only Risser 0 patients, regardless of TRC being open or closed, demonstrated significant growth in instrumented segments (range 12–18 mm). Statistically significant asymmetric growth (concave > convex) was never observed. No significant differences in UIV-LIV angle between first standing and final follow-up radiographs were observed, but patients with an open TRC had an insignificant improvement of 4° while Risser 2–5 patients had an insignificant worsening of 2°.

During VBT, a tensioned cable connected to vertebral implants on the convex side partially corrects the deformity intra-operatively and in theory modulates remaining growth post-operatively [7, 8, 20–24]. Growth modulation of VBT relies on the Hueter-Volkmann law, which states that compression on the convex side of vertebra following VBT slows growth, but clinical evidence of this principle is limited following VBT surgery [24, 25].

A retrospective study of 51 patients (Sanders 2–4) with 2 years follow-up reported the UIV-LIV angle improved from 46° ± 11° pre-operatively to 17° ± 11° at 2 years follow-up. Unfortunatley the study did not report if the angle changed from immediate post-op to final follow-up, so one cannot conclude that UIV-LIV angle was influenced by growth modulation following VBT [26]. In our study, there was no significant improvement or worsening of the UIV-LIV angle between first standing and final follow-up radiographs. Although significant vertebral growth was demonstrated for Risser 0 patients over the instrumented vertebrae, asymmetric growth was not observed.

McDonald et al. reported on change in height of individual instrumented vertebra and discs following VBT. They found the convex discs decreased in height by a mean of 1.2 ± 1.9 mm while concave discs did not change from instrumentation at 2 years follow-up. Differential growth was observed in instrumented vertebra, in which the concave side grew 2.0 ± 2.2 mm compared with 1.5 ± 2.3 mm on the convex side (p < 0.001). It is notable that one standard deviation of measurement variability is greater than the average change in height. In this study, the average patient had surgery amongst 5 vertebra and 4 discs, so the total variability/error added over 9 discrete measurements can be quite large. Unfortunately the change in total height of instrumented segments were not reported, so the net change in convex versus concave instrumented height is unknown. In contrast, the current study measures the total height of the UIV-LIV segment, which we suspect is subject to less additive error than measuring individual segments of vertebra and disks.

The McDonald study design has other major differences with the current study. First, their study excluded patients with greater than 35° instrumented major curve at final follow-up, stating the study only wanted to focus on successful cases of VBT. However, our study included patients with all ranges of UIV-LIV angles at final follow-up, so we report on a true mean for all patients at various stages of growth. Second, the study by McDonald et al. reported growth at 2 years following VBT, whereas our study reported growth at an average of 3.8 years following VBT – nearly double.

In summary, we found that tethered segments grew significantly (Table 2) for Risser 0 patients, but this growth was not asymmetrical and did not show significant curve correction. The data in this study rejects our 2 hypotheses: (1) VBT was not associated with asymmetric (concave > convex) increases in height over instrumented vertebra and (2) instrumented Cobb angle did not improve following VBT surgery. Although instrumented segments grew for Risser 0 patients undergoing VBT, the lack of significant improvement in UIV-LIV angle makes one wonder whether the benefits outweigh some of the reported risks of the surgery. When reporting on 17 Risser 0 patients (94% TRC open) undergoing VBT, Newton et al. found a 23% overcorrection rate, with 4 patients requiring revision surgery [27]. Another study found a 19% overcorrection rate, with all occurring in Sanders ≤ 3 [28]. Additionally, a recent systematic review found 21.3% of patients undergoing VBT to experience a confirmed or suspected broken tether [29], the clinical significance of which is unclear at this point. It is evident we are still learning more about this novel approach to pediatric deformity.

There were several limitations to this study. First, since we excluded patients with tether breakages, the growth and curve correction in such patients are unknown. Second, it is possible that there was a change in vertebral growth and angles outside of the instrumented vertebrae, but that is not the subject of this focused study. Third, there was no control group of patients with normal or untreated scoliotic spines for direct comparisons. Fourth, due to the new nature of this technique, there are limited patients which fit the strict inclusion criteria of 2 years follow-up. Having more patients could yield different results. Fifth, while the mean follow-up was 3.8 years after surgery, additional spinal growth may be seen past this point. Sixth, as VBT is still a new procedure, techniques amongst included surgeons may have varied considerably and been partially responsible for our findings. Differences may range from the use of a video-assisted thorascopic approach to how the tether is tensioned. Lastly, measurement technique when evaluating radiographs can also introduce some variability in results. We believe we have minimized this by measuring the height of the total instrumented segment instead of each individual instrumented level. Since a height measurement requires a reviewer to mark two discrete points on the radiograph, regardless of the magnitude of height, making a measurement of the entire construct reduces the proportion of height that expected variability is responsible for. A recent study assessing the reliability of height measurements for vertebral bodies following VBT found an interobserver agreement ranging from good to moderate, which is already acceptable [30]. However, we believe that the interobserver agreement would have been even higher if using the methodology of the current study.

## Conclusion

At a mean of 3.8 years following VBT, 33 Risser 0 patients demonstrated significant growth in the instrumented segment, though there was no difference between concave or convex growth, even for patients with open TRC. The instrumented Cobb angle in patients with open TRC had an insignificant improvement of 4°; and those Risser 2–5 had an insignificant worsening of 2° following VBT.

## Data Availability

The datasets generated and analyzed during the current study are available from the corresponding author on reasonable request.
